# Recellularization of a novel off-the-shelf valve following xenogenic implantation into the right ventricular outflow tract

**DOI:** 10.1371/journal.pone.0181614

**Published:** 2017-08-01

**Authors:** Ryan S. Hennessy, Jason L. Go, Rebecca R. Hennessy, Brandon J. Tefft, Soumen Jana, Nicholas J. Stoyles, Mohammed A. Al-Hijji, Jeremy J. Thaden, Sorin V. Pislaru, Robert D. Simari, John M. Stulak, Melissa D. Young, Amir Lerman

**Affiliations:** 1 Department of Cardiovascular Diseases, Mayo Clinic, Rochester, Minnesota, United States of America; 2 Department of Internal Medicine, University of Kansas School of Medicine, Kansas City, Kansas, United States of America; 3 Division of Cardiovascular Surgery, Mayo Clinic, Rochester, Minnesota, United States of America; Brigham and Women's Hospital, Harvard Medical School, UNITED STATES

## Abstract

Current research on valvular heart repair has focused on tissue-engineered heart valves (TEHV) because of its potential to grow similarly to native heart valves. Decellularized xenografts are a promising solution; however, host recellularization remains challenging. In this study, decellularized porcine aortic valves were implanted into the right ventricular outflow tract (RVOT) of sheep to investigate recellularization potential. Porcine aortic valves, decellularized with sodium dodecyl sulfate (SDS), were sterilized by supercritical carbon dioxide (scCO_2_) and implanted into the RVOT of five juvenile polypay sheep for 5 months (n = 5). During implantation, functionality of the valves was assessed by serial echocardiography, blood tests, and right heart pulmonary artery catheterization measurements. The explanted valves were characterized through gross examination, mechanical characterization, and immunohistochemical analysis including cell viability, phenotype, proliferation, and extracellular matrix generation. Gross examination of the valve cusps demonstrated the absence of thrombosis. Bacterial and fungal stains were negative for pathogenic microbes. Immunohistochemical analysis showed the presence of myofibroblast-like cell infiltration with formation of new collagen fibrils and the existence of an endothelial layer at the surface of the explant. Analysis of cell phenotype and morphology showed no lymphoplasmacytic infiltration. Tensile mechanical testing of valve cusps revealed an increase in stiffness while strength was maintained during implantation. The increased tensile stiffness confirms the recellularization of the cusps by collagen synthesizing cells. The current study demonstrated the feasibility of the trans-species implantation of a non-fixed decellularized porcine aortic valve into the RVOT of sheep. The implantation resulted in recellularization of the valve with sufficient hemodynamic function for the 5-month study. Thus, the study supports a potential role for use of a TEHV for the treatment of valve disease in humans.

## Introduction

The most common valvular heart disease affecting morbidity and mortality lies in the aortic valve. In the United States alone, an estimated 20,000 infants are affected by congenital heart disease with another 51,000 adults over age 65 are affected by age-related calcific aortic stenosis and insufficiency [1]. Currently, there are two approaches for AVR: mechanical and bioprothetic prostheses [2]. Mechanical heart valves are susceptible to infection and require life-long anticoagulants, while bioprothetic valves have limited durability and are frequently subjected to replacement [3]. Moreover, neither of the available options will be able to grow within a pediatric patient [4]. Current research on valvular heart repair has focused on tissue-engineered heart valves (TEHV) because of its potential to grow similarly to native heart valves [5].

The current applications of bioprothetic valves involve gluteraldehyde-fixation of porcine or bovine tissue [2]. This method is widely used in medicine for its ability to mask xenoantigens; however, this approach is susceptible to calcification, valve deterioration, and inability to recellularize in an *in vivo* environment [3]. Gluteraldehyde-fixation preserves tissue at the expense of the living cell, and therefore is unable to grow within its intended population. Although these prosthetic approaches have been an immediate solution to AVR, growth with the patient, valve durability, and the need for anticoagulation therapy continue to be issues that need resolving.

A process known as decellularization exposes biological tissue to various chemical, biologic, or physical processes to remove cellular components without disrupting the extracellular matrix [6]. This is done to minimize antigenicity and maintain mechanical integrity and signaling factors found in native tissue. The TEHV in this study uses a non-fixed decellularized xenograft because it is a platform for host cells to recellularize, repair, and remodel tissue [7]. The goal was to establish a decellularization method that would remove the vast majority of cellular proteins, DNA, and other antigenic fragments from a xenograft prior to implantation [8, 9]. While decellularization attempts to make constructs less antigenic, sterilization is also needed to reduce bacterial, fungal, and viral contaminants that lead to infection. Ultimately, sterilization will be required for off-the-shelf clinical implementation. We have recently reported the use of several sterilization methods for porcine aortic valves and demonstrated that the use of scCO_2_ was preferable for its ability to sterilize and maintain sufficient tissue mechanical properties [10, 11]. The removal of cellular debris through decellularization coupled with sterilization of tissue would prevent both an inflammatory and immune response that would allow for exploration of *in vivo* recellularization studies. The present study was designed to test the hypothesis that a non-fixed decellularized porcine aortic valve sterilized with scCO_2_ and implanted into the RVOT of sheep would recellularize in an *in vivo* environment.

## Methods

### 1. Ethical statement

All sheep utilized were housed together at the Mayo Clinic Institutional Hills Farm and acclimatized to the environment for two weeks prior to scheduled procedures. Standard of care was approved by Mayo Clinic’s Institutional Animal Care and Use Committee (IACUC Protocol# A38813-13) throughout the duration of the study.

### 2. Procurement, decellularization, and sterilization of porcine valve scaffolds

Freshly harvested whole porcine hearts were randomly selected and obtained from a local abattoir (Hormel Food Corporation, USA). The porcine myocardium was dissected and trimmed leaving the aortic root, aortic valve cusps, as well as the anterior cusp of the mitral valve intact. The decellularization process involved 1% Sodium dodecyl sulfate (SDS), DNase, and diH_2_O with constant agitation. An additional exposure and wash with 2% DNase, Tris buffer, MgCl_2_, 1% peroxyacetic acid (PAA), and phosphate buffered solution (PBS) was performed. The decellularized valves were sterilized using supercritical carbon dioxide (NovaSterilis, Inc., USA) [11]. The detailed decellularization and sterilization process parameters are outlined in our previous work [10].

### 3. Animals

Five healthy male and female juvenile polypay sheep (Neaton’s Polypay, MN) were randomly selected with an average arrival weight of 37 kg ± 2.3 kg. A sample size of 5 was selected to assess valvular performance *in vivo*. Once feasibility is demonstrated, additional animals with a larger sample size will be evalulated in the aortic position. Each sheep was evaluated individually by a Mayo Clinic Veterinarian prior to study. Preoperative assessment of the sheep involved a transthoracic echocardiogram (TTE), which revealed no abnormalities.

### 4. Surgical implantation and *ex vivo* tissue analysis

Decellularized and steriled porcine aortic valves were placed into the right ventricular outflow tract in both the annulus and sinotubular junction locations of juvenile polypay sheep for five-months. The anaesthesia used were diazepam 0.4–0.5mg/kg, ketamine 7.5 mg/kg, morphine sulfate preservative-free (10 mg/ml, 1 ml) 0.5 mg/kg, propofol 3-5mg/kg to effect, and isoflurane 2–3.5% continuous until the procedure was completed. Each sheep, approximately 4-months old and weighing 30 kilograms, were put on a normothermic cardiopulmonary bypass without arrest or aortic clamping. The operation was executed via a left thoracotomy and incision above the RVOT was performed [12]. Native pulmonic cusps were removed. Each sheep was then given intercostal nerve blocks prior to closure. Ceftiofur was given intramuscularly prior to their procedure and given again on the 3^rd^ day post op. Cefazolin was also given intravenously 15 minutes prior to incision. All sheep studied underwent a transthoracic echocardiogram (TTE) before and after incision to study valvular size, valvular hemodynamics, and right ventricular function. In addition, post-bypass weaning epicardial echocardiography was done to assess valve dynamics and ventricular function [12]. Animals were housed indoors during the first post-operation week and given warfarin TID for 2 days. Vital signs were routinely monitored; blood was drawn for complete blood count (CBC) and chemistry studies, and the sheep were then transferred to the Mayo Clinic farming facility for monthly follow-ups. Prior to sacrifice, animals were given a final 5-month TTE, blood draw, and a right heart catheterization to accurately assess the implanted valve gradient. Sacrifice was performed with pentobarbital (Sleepaway) 250 mL vial.

### 5. Determination of *in vivo* valvular hemodynamics

Images of the porcine aortic valves in the RVOT were observed using a TTE approach. The TTE allowed for observance of the hemodynamics and structural integrity of the valves implanted in the sheep with vigilance specifically on stenosis and insufficiency [13]. Serial systolic mean gradients, measured by simplified Bernoulli equation (Pressure gradient = 4*blood cells systolic flow velocity across valve^2^) [14] were calculated at the end of the initial-week after transplantation and once a month for 5 months. Study animals were imaged from a left parasternal window while in a standing position. Short axis images of the mid and basal left ventricle were performed with 2D imaging. The pulmonary valve xenograft was evaluated by 2D, color Doppler, and spectral Doppler imaging from a basal short axis view. Supportive findings for prosthetic stenosis included increased leaflet thickness and reduced leaflet excursion visualized by 2D imaging, elevated systolic peak jet velocity or systolic mean gradient by continuous wave Doppler, or a progressive increase in systolic peak jet velocity or mean gradient observed on serial studies [15]. Prosthetic valve regurgitation was judged using an integrated approach; components of the evaluation included the regurgitant jet width by color Doppler, jet penetration depth into the right ventricular outflow tract by color flow Doppler, and the regurgitant jet density and deceleration rate by continuous wave Doppler [15]. Rapid deceleration of the spectral Doppler signal that reached the baseline before the end of diastole was considered consistent with severe regurgitation.

A hemodynamic right heart catheterization was performed at 5 months immediately prior to sacrifice. A Swan-ganz pulmonary artery catheter (Edwards LifeSiences, Irvine, CA) was inserted via the internal jugular vein and advanced into the pulmonary arteries. A mean right atrial pressure > 6 mmHg was considered high. The peak-to-peak valve gradient was calculated by subtracting the peak right ventricular systolic pressure from the peak systolic pulmonary artery pressure. Pulmonary hypertension was defined as a mean pulmonary artery pressure (PAP) ≥ 25 mmHg. The left sided filling pressures were assessed using pulmonary capillary wedge pressure (PCWP) (>12 mmHg considered high).

### 6. Biomechanical properties of porcine aortic valves: Tensile testing

One full cusp from each aortic valve was used for tensile property testing. Each valve (n = 5) was stored in PBS at 4°C for no more than 5 hours before processing. Cusps were cut into rectangular 8mm (height) and 2mm (width) shapes using a razor blade. Collagen fibers were aligned circumferentially and digital calipers were used to measure the thickness at the center. Three thickness measurements were taken and the average was used for data analysis. Samples were mounted into the tensile tester (Bose ElectroForce 3200, Bose Corporation, Eden Prairie MN) [1, 8, 10, 16]. The uniaxial tensile test was performed at crosshead displacement speed of 0.1667 mm/s per ASTM standards [17] (ASTM F2150-13). The ultimate strength and stiffness were calculated from the resulting stress strain graph using the load vs displacement data using the WinTest Bose software. It should be noted that aortic valve cusps experience complex bidirectional flexure in vivo [18, 19]. To simplify the problem, we focused on bending in the circumferential direction only, as it is the major curvature change in cusps [20, 21].

### 7. Histology and immunohistochemistry

Portions of explanted tissue were removed, sectioned, and embedded using an optimal cutting temperature compound (OCT) methylmetacrylate and frozen in liquid N_2_. The tissues included were ¼ of the aortic valve cusp, sinus, and both distal/proximal ovine/porcine anastomosis. Various antibodies were used for immunohistochemical (IHC) staining to confirm recellularization of the explanted tissue. CD31 (Abbiotec, San Diego, CA) and von Willibrand factor (Abcam, Cambridge, UK) antibodies stained for endothelialization; α-SMA (Sigma, St. Louis, MO) and Vimentin (Abcam) antibodies tested for the presence of interstitial-like cell phenotypes [22–25]. In order to test the existence of α-gal epitope, decellularized tissue was stained using the α-Gal Epitope (Galα1-3Galβ1-4GlcNAc-R) monoclonal antibody M86 (Enzo Life Sciences, Farmingdale, NY) prior to implant. Tissue samples were immune labeled with the Dako EnVision System-HRP, blocked with the Dako peroxidase solution (Aligent, Santa Clara, CA), incubated in primary antibody overnight at 4°C, and stained with a species-matched secondary antibody (biotinylated mouse anti-mouse / rabbit anti-rabbit Ig F [ab’]2 fragments; Dako). The bound antibody was visualized using Sigma Fast 3, 3-diaminobenzidine (DAB) and the sections were counterstained, dehydrated, and mounted [26]. Images were captured at 10x and 20x magnifications using light microscopy. In addition to IHC staining, samples underwent Haemotoxylin-Eosin (H&E), Brown and Brenn Gram, Periodic Acid Schiff (PAS), Von Kossa, Alizarin red, Picrosirius red, and Masson Trichrome stains to observe DNA, microbial contaminants, polysaccharides, calcification, and fibrosis; respectively [25].

### 8. Statistical analysis

Continuous variables are presented as mean ± SD or median (25th, 75th percentiles) depending on the normality of distribution. Comparisons of baseline and follow-up systolic mean gradient of the implanted valve were measured using the nonparametric Wilcoxin signed rank test. The differences in tensile properties of the five explanted valves were compared to decellularized and native porcine valves using Wilcoxin/Kruskal-Wallis test. Statistical significance was defined as a 2-tailed *P* value of less than 0.05. Statistical analyses were completed utilizing SAS 9.4 (SAS Institute Inc, Cary, North Carolina).

## Results

### 1. Physical examination and laboratory analysis

Five decellularized valves with sterilization were implanted into the RVOT of 5-juvenile sheep. There were no signs of illness following surgical implantation, and all five sheep survived the entire 5-month study with functioning valves and no perioperative complications. There were no clinical signs of infection or inflammation at the surgical incision site. All five animals were euthanasied at the 5-month study end point. Vital signs, including temperature and blood pressure, were within normal limits over 5-months. Throughout the study, weights and labs were routinely performed. The average arrival weight was 37 kg ± 2.3 kg (Table 1), which increased to 60 kg ± 3.5 kg at time of sacrifice. Laboratory analysis showed white blood cell, red blood cell, hematocrit, and lymphocyte counts within normal limits (Table 2).

**Table 1:** Average weight of sheep reported in mean and standard deviation.

Pre-Op	1-month	3-months	5-months
36.9 Kg (2.3)	42.3 Kg (2.4)	49.4 Kg (1.7)	60.4 Kg (3.5)

The average arrival weight was 37kg ± 2.3kg; at time of sacrifice, the average weight had doubled in size to 60 ± 3.5 kg.

**Table 2:** Laboratory analysis reported in mean and standard deviation.

	Pre-Op	5-months
**WBC (Normal 4–12 x 10**^**9**^**/uL)**	6.1 (1.8)	7.9 (2.2)
**Lymphocytes (Normal 2–9 x 10**^**9**^**/uL)**	5.8 (1.8)	7.1 (2.4)
**RBC (Normal 9–15.8 x 10**^**12**^**/uL)**	10.3 (0.6)	9.4 (1.0)
**HCT (normal 27–45%)**	26.4 (1.8)	26.0 (2.0)
**Platelets (Normal 250–750 x 10**^**3**^**/ μL)**	228.0 (42.0)	282.4(73.0)

Laboratory analysis in pre-operative period and at 5-months showed white blood cell, red blood cell, hematocrit, and lymphocyte counts within normal limits.

### 2. Determination of in vivo valvular hemodynamics

**Fig 1:**
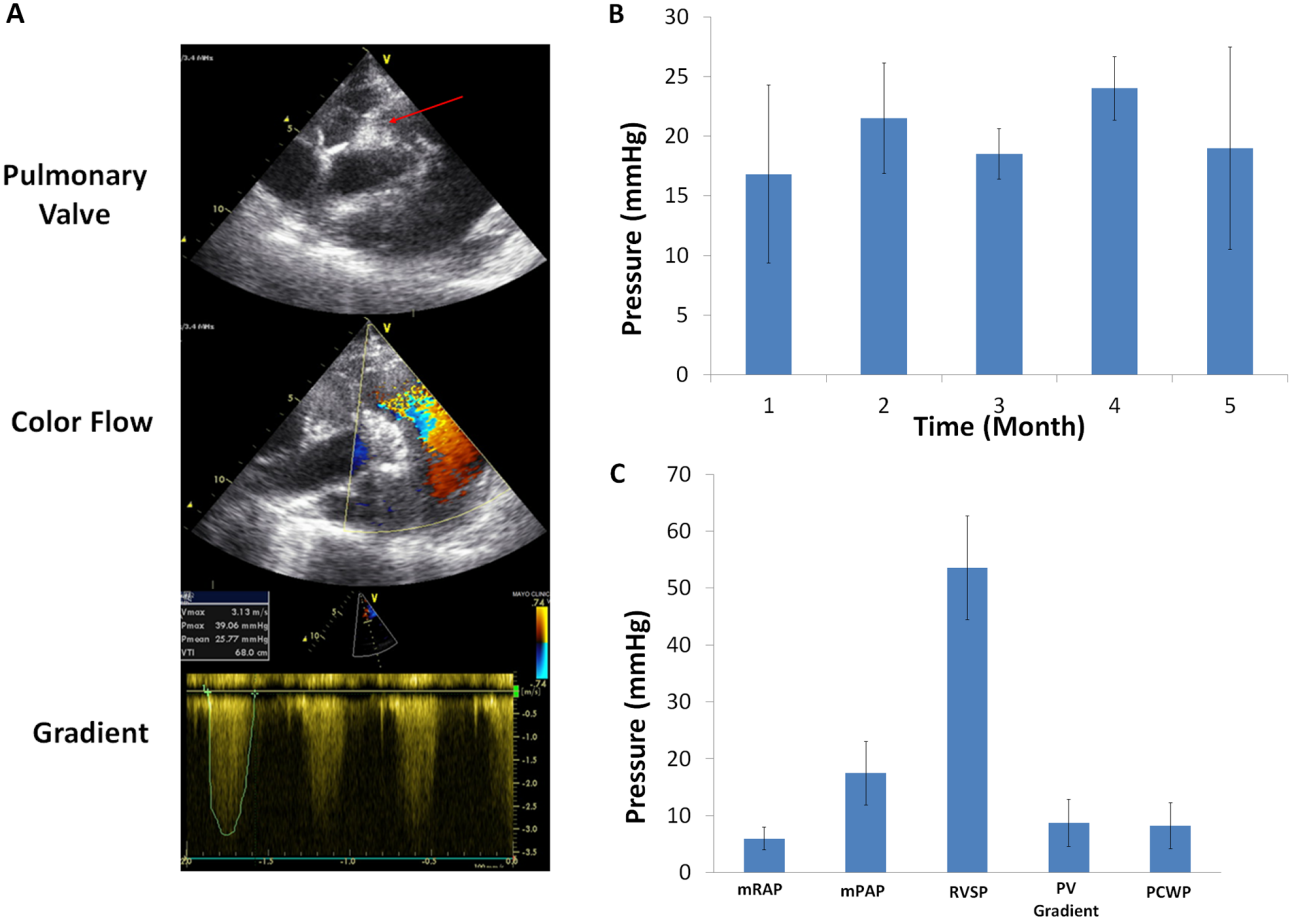
Determination of i*n vivo* valvular hemodynamics. (A) Representative echocardiographic image at 3-months. The aortic valve cusps were free of fibrosis or thickening arrow indicates aortic valve cusps. The Doppler flow confirms no valve stenosis, and gradient was measured. (B) Systolic gradients across the pulmonic valves over time measured using continuous wave Doppler echocardiography. The mean pressure gradient was elevated at one month but remained unchanged over the course of the study (P = 0.25 for mean gradient at 1- vs 5- months). (C) Hemodynamic measurement taken immediately prior to explant using invasive pulmonary artery catheters. mRAP, mean right atrial pressure; mPAP, mean pulmonary artery pressure; RVSP, right ventricular systolic pressure; PV, pulmonic valve peak to peak gradient; PCWP, pulmonary capillary wedge pressure.

*In vivo* valve performance was evaluated with echocardiographic imaging and right heart catheterization. The mean systolic pressure gradient was elevated at 1-month, but it did not change significantly over the entirety of the study (16.8 mmHg ± 7.5mmHg at 1 month vs 19 mmHg ± 8.5 mmHg at 5 months; P = 0.25). The implanted valves appeared to have normal mobility on 2D TTE with normal cusps excursion. The valves were not thickened or calcified and were free from vegetation. Continuous wave and color flow Doppler showed moderate valvular insufficiency assessed by rate of deceleration and the depth of regurgitant jet penetration into the RVOT, respectively (Fig 1). These assessments were made by two independent cardiologists using guidelines outlined by the American Society of Echocardiography and Standards Committee and the Task Force on Prosthetic Valves [15, 27].

The peak to peak xenograft valve gradient measured by right heart catheterization was 8 mmHg ± 4 mmHg. The mean right atrial pressure (RAP) and mean PAP were normal at 6 mmHg± 2 mmHg and 17 mmHg ± 6 mmHg, respectively.

### 3. Histology and immunohistochemistry

**Fig 2:**
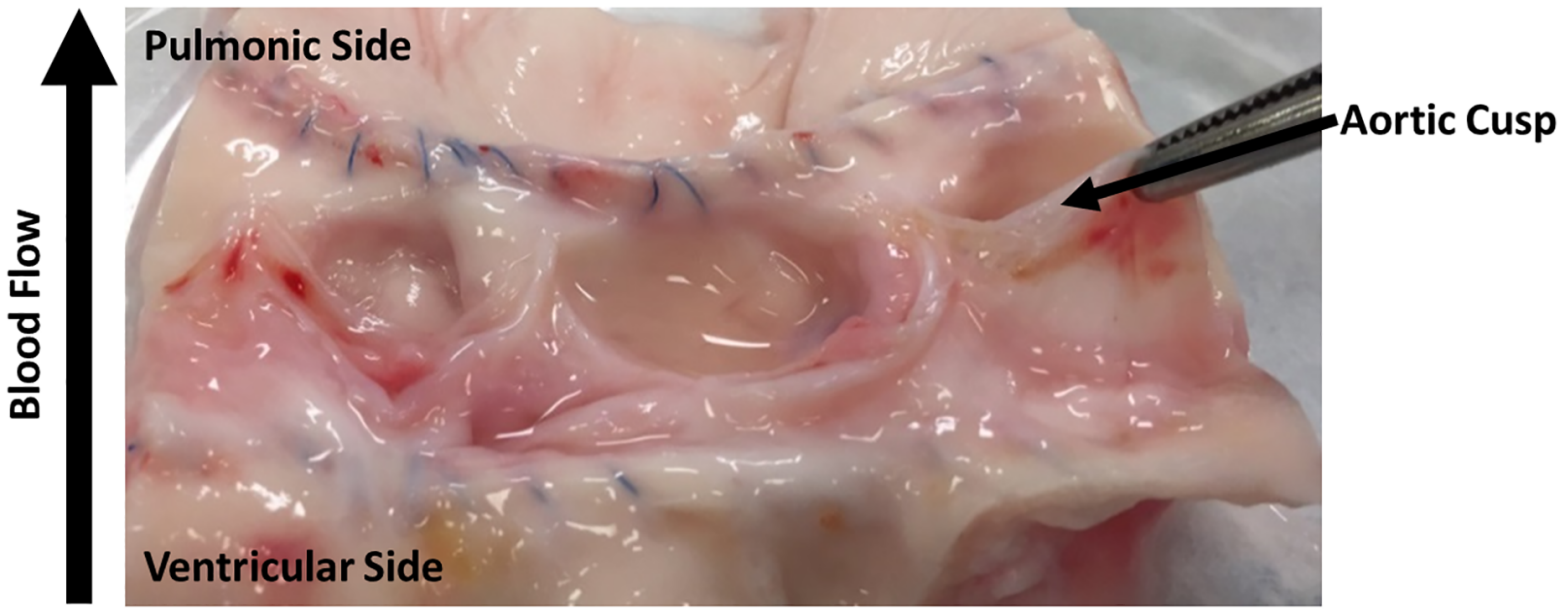
Gross examination. Explanted aortic valves showing aortic cusp without evidence of thrombosis, increased thickness, or calcification. Explant taken immediately after 5-month sacrifice and cross-sectioned to show anatomical features.

**Fig 3:**
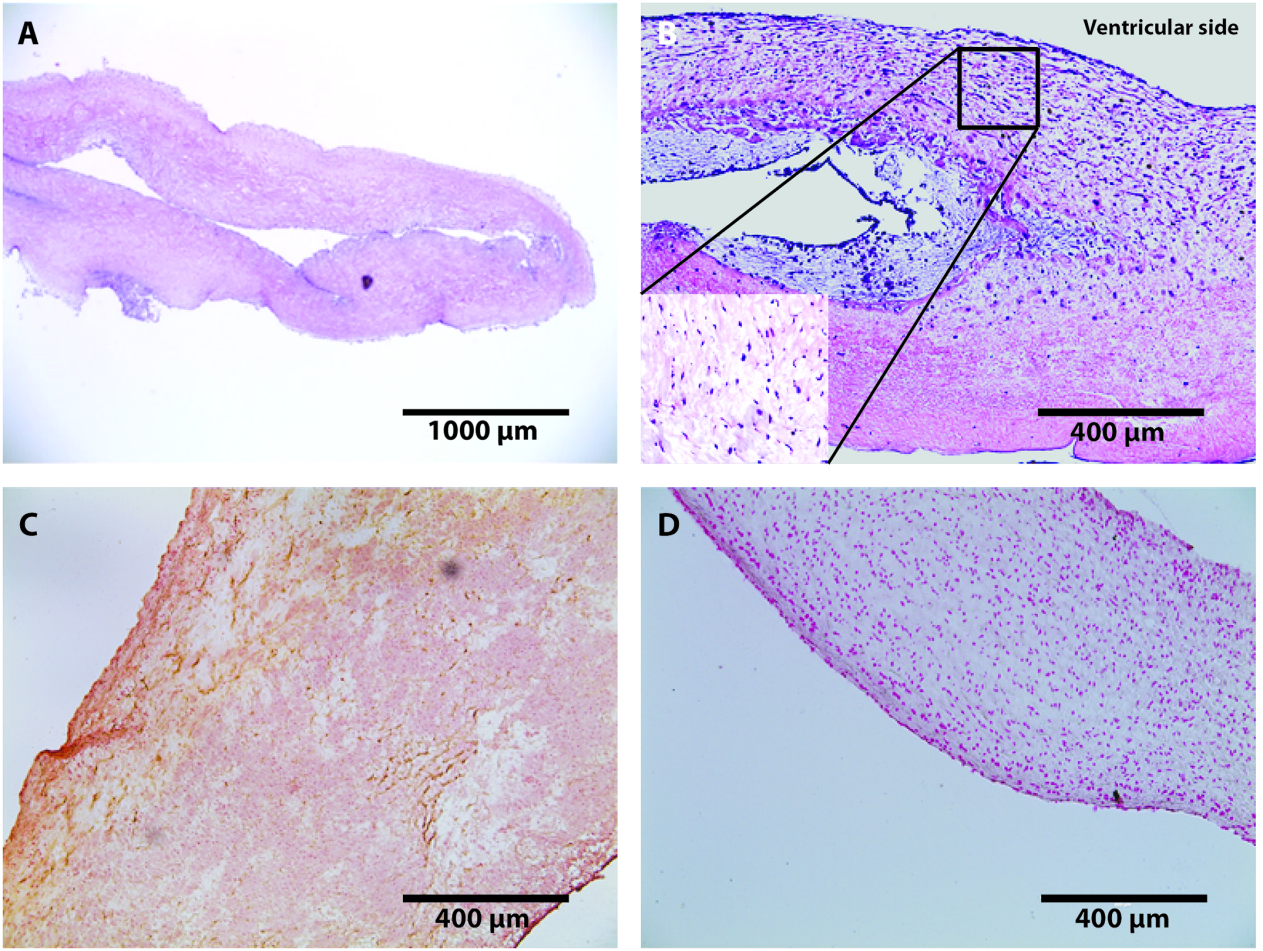
Staining of porcine aortic cusps post-explant at 5-months. H&E staining representative (A) Histology section of decellularized porcine cusp prior to implantation (scale: 1000 μm). Image shows ECM intact without presence of nuclear cellular material. (B) Recellularized Aortic cusp of sheep (scale: 400 μm). Image shows intact ECM with infiltration of host-derived cells as shown in the magnified image within stain B at (20x). (C) Alizarin Red; characterization of aortic cusps post-explant at 5-months representative images (scale: 400 μm) showing absence of calcification on tissues, (D) Von Kassa; characterization of aortic cusps post-explant at 5-months representative images (scale: 400 μm) showing absence of calcification on tissues. In the case of calcification, stains C and D would show hyderdense dark areas.

**Fig 4:**
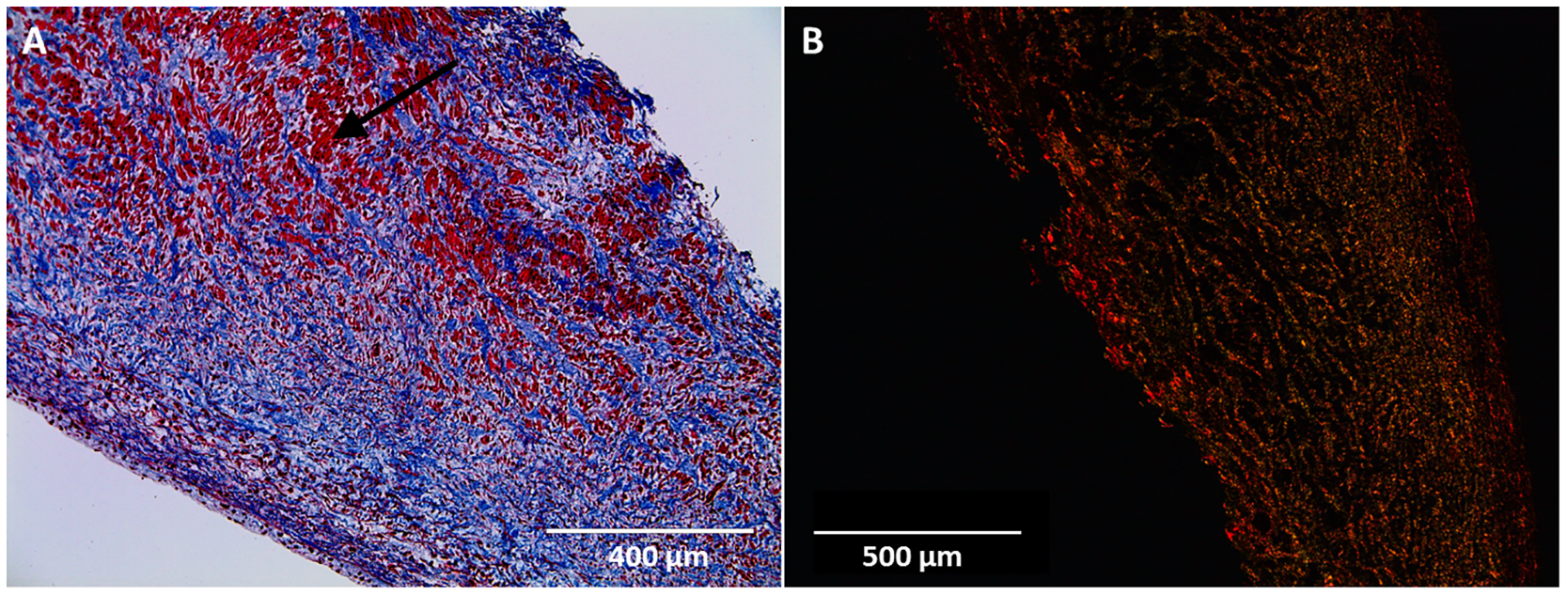
Masson’s trichrome and picrosirius red staining of porcine cusps 5-months post-explant. A) Masson’s trichrome stain (scale: 400 μm) shows ECM collagen in blue (Black arrow). Pink/Red areas are representative of nuclei and cytoplasm. B) Picrosirius Red stain (scale: 500 μm) under polarized light characterizes collagen formation.

**Fig 5:**
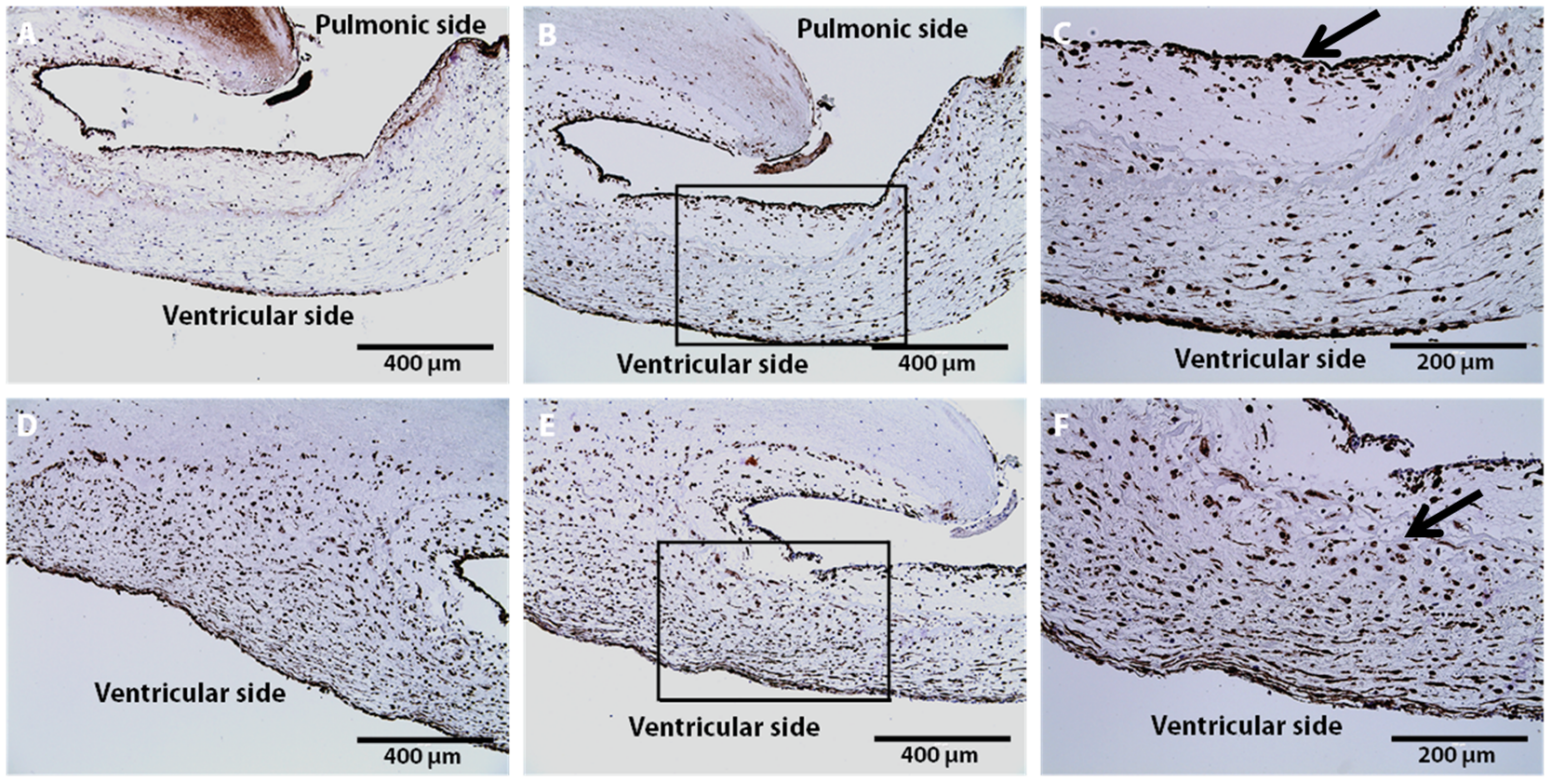
Immunohistochemical characterization of aortic cusps post-explant at 5-months. Recellularization of VICs and VECs found in the aortic cusps (scale: 400 μm). Moderate endothelialization of aortic cusps on the pulmonic side observed using A.) VWF and B.) CD31 antibody. C) Representative magnification of box found in (B) showing VEC infiltration (20x) along the periphery of cusp denoted by black arrow. Infiltration of myofibroblast-like cells originating from the ventricular side observed using D.) Vimentin and E.) α-SMA antibodies. F) Representative magnification of box found in (E) showing VIC infiltration (20x) seen inside the cusp tissue denoted by black arrow.

Gross examination of valve cusps demonstrated inflammation and the absence of thrombosis (Fig 2). Damage that was observed include cusp retraction inside the aortic wall, adhesions, and perforations on one cusp. At explant, H&E staining was performed on one randomly selected cusp from each sheep. Fig 3 shows that the extracellular matrix (ECM) remained intact and cellular infiltration was seen in all samples. These results were compared to an H&E stain of a decellularized xenograft prior to implant. The ECM remained intact and looked similar to native ovine post explant. Brown and Brenn Gram staining and Periodic Acid-Schiff staining was negative for microbial and fungal contaminants, respectfully. Culture swab results showed no growth on the valve. Masson Trichrome and Picrosirius Red was performed and provided a clear image of the collagen structure which indicated no fibrosis (Fig 4). Alizarin Red and Von Kossa stains showed no cusp calcification. In addition to evaluating the cusps, we evaluated the the aortic vall of our explanted constructs using H&E staining. Immunohistochemical staining assessed both absence of α-gal epitope and the recellularization potential of one randomly selected cusp from each sheep. There was VEC monolayer formation observed throughout the periphery of our cusps in all five sheep as seen in vWF and CD31 antibody stains (Fig 5). Assessment of cell infiltration using alpha-SMA and vimentin antibodies showed that there was a reasonable amount of myofibroblast-like cell infiltration found in the cusps. Absence of α-gal epitope was shown using monoclonal M86 anti-α-gal primary antibody.

### 4. Biomechanical properties of porcine aortic valves: Tensile testing

**Fig 6:**
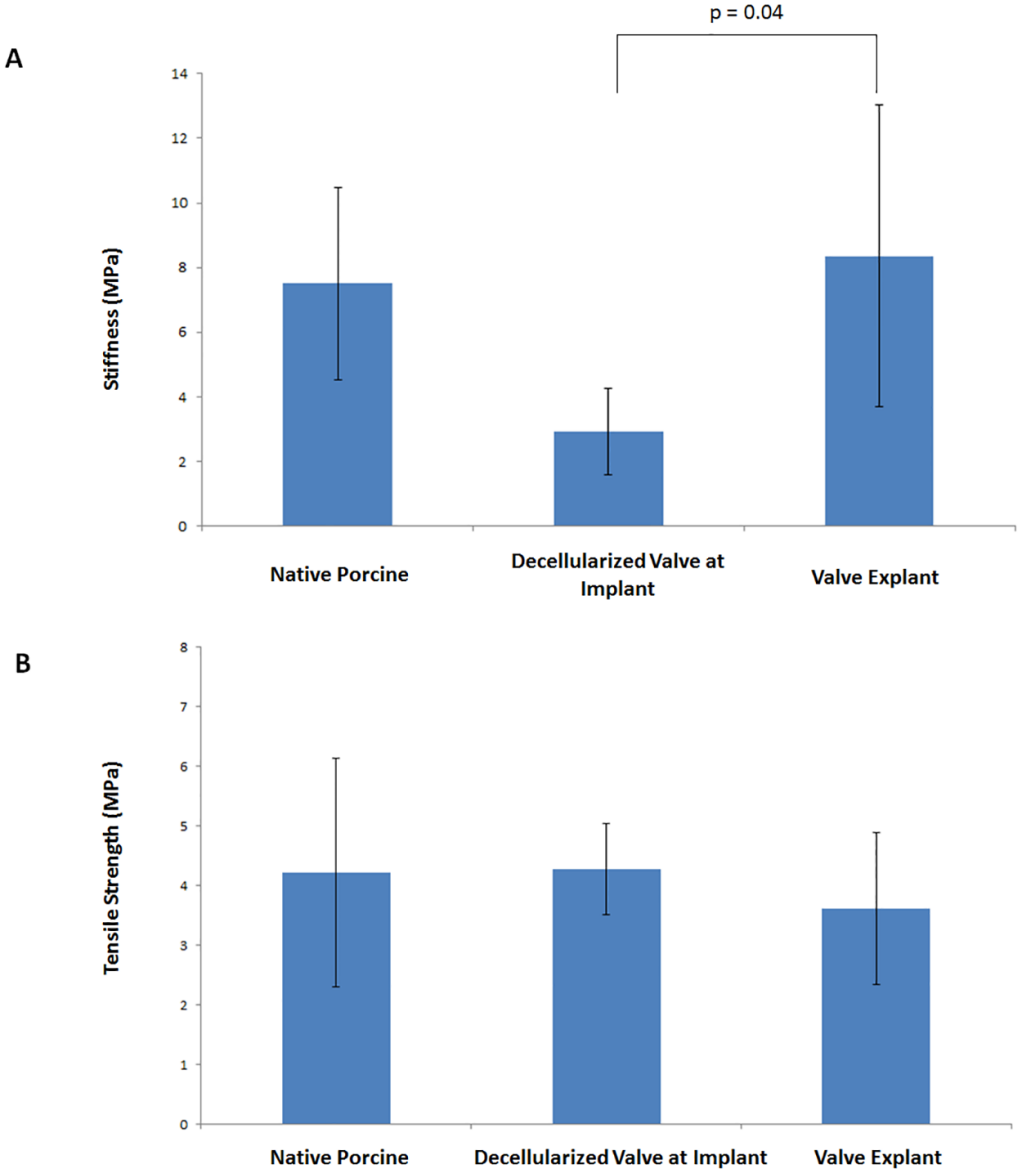
Mechanical properties. (A.) Stiffness comparison of native porcine, decellularized porcine with sterilization, and recellularized porcine explant after 5 months in vivo. (B.) Ultimate tensile strength comparison of native porcine, decellularized porcine implant with sterilization, and recellularized porcine explant after 5-months in vivo.

Uniaxial mechanical testing was performed on one randomly selected cusp from each explanted sheep. The specimens were tested in the circumferential direction (n = 5). The stress strain results demonstrated an increase in stiffness (8.4 MPA ± 4.7 MPA in valve explant vs 2.9 MPA ± 1.3 MPA in the decellularized and sterilized processed valve; P = 0.04), while the ultimate tensile strength remained similar (3.6 MPA ± 1.3 MPA in valve explant vs 4.3 MPA ± 0.8 MPA in decellularized and sterilized processed valve; P = 0.99). The stiffness and strength of valves at explant were similar to native porcine valves (Fig 6). The valve processing significantly decreased the stiffness, and recellularization that occurred during the implantation period regained the native stiffness. The mean and standard deviation for cusp thickness was observed to be 0.38 (0.4) for native, 0.26 (0.3) for implant processed valves, and 0.38 (0.3) for explanted valves.

## Discussion

The current study demonstrated that a decellularized porcine aortic heart valve with sterilization maintained tissue integrity and allowed ovine cell infiltration in an *in vivo* environment. The current study shows successful 1) trans-species implantation, 2) a decellularized processed xenograft, 3) valve off-the-shelf sterility, and 4) recellularization of host cells. Thus, the study supports a potential role for use of a TEHV for the treatment of valvular heart disease in humans.

The sheep’s valvular anatomy, physiology, function, and coagulation cascades are comparable to humans making it a favorable cardiovascular research model [28–30]. In our study, five decellularized porcine aortic valves were implanted into the RVOT of sheep. Previous work has demonstrated the complexity of a trans-species implantation due to the interaction between primate anti-Gal antibody and the α-Gal epitope (Galα1-3Galβ1-4GlcNAc-R) expressed in porcine tissues [31]. This interaction of anti-Gal antibody and α-Gal epitope has been one of the biggest obstacles in xenograft transplantation. It has been observed that the α -Gal-specific IgM-antibodies found on the cell surface of primates may lead to complement activation and hyperacute rejection [32]. Although this is a large obstacle in porcine-to-human xenograft implantation, our study utilized an ovine model which does not have this anti-Gal antibody and α-Gal epitope interaction. It is entirely possible to process a xenograft that removes this antigen from porcine tissue allowing for implantation into a primate model [33]. The main goal of our study was to successfully implant a processed xenograft into a model that has comparable hemodynamic functionality found in humans. This was achieved in our study with sheep.

Invasive and non-invasive hemodynamics in sheep after porcine aortic valve implantation in the RVOT was evaluated. The xenograft valves demonstrated normal mobility with normal leaflet excursion on 2D TTE throughout the study. This was further supported by the absence of thrombosis or calcifications on gross inspection and immunohistochemistry of the valves explant. However, the mean systolic gradients across the xenograft valves were elevated throughout the study. Increasing flow across the xenograft valves secondary to significant valvular insufficiency is the likely responsible mechanism for the elevated mean systolic gradient measurements by 2D TTE CW Doppler, especially in the absence of valvular stenosis, infection, or high metabolic demand such as anemia. The valvular insufficiency observed was likely related to cusps retraction to the arterial wall, which was observed frequently in other decellularized valve expirements [28]. Overall, the xenograft valves had adequate hemodynamics as demonstrated by normal filling pressures and normal pulmonary pressures at the time of explant.

The decellularization process can produce a cell-free biological scaffold, but production of such a construct comes at the expense of disrupting the ECM [6, 8, 9, 34]. The decellularization process is dependent on tissue cellularity, density, lipid content, and thickness [6]. A chemical approach for decellularization was used in our studies because biological and physical approaches have not been fully explored with large animals. Previous studies have determined that SDS was suitable for removing cellular debris from specific xenograft tissues including aortic valves [6]. However, prolonged exposure to any type of detergent-based solution may lead to loss of tissue architecture. As a result, an optimized decellularization process was implemented to remove cellular remnants while preserving as much of the ECM as possible. Our decellularized valvular construct showed no cellular presence, and this was confirmed using H&E and DAPI staining [10]. The absence of cellularity from the porcine aorta was essential because it allowed us to establish a construct that was free from any antigens that would be responsible for a graft-vs-host reaction. The importance of removing residual donor cells from the aortic valve root and cusps using a chemical-based decellularization technique have been explored by many groups [5, 25, 33, 35–37]

In order to introduce our valvular construct clinically, it is critical to integrate a sterilization step. An off-the-shelf sterilized valve would be practical and safe for clinical applications when compared to a decellularized valve that requires cell seeding and aseptic conditioning. In prior experiments, our group demonstrated that scCO_2_ removed microorganisms and maintained tissue integrity after decellularization [10]. Presence of microbial contaminants such as bacteria, viruses, or fungus can accelerate decomposition of a biological construct. In our present study, we have shown that a decellularized valve with sterilization can be implanted resulting in host-cell infiltration and maintenance of tissue integrity with no evidence of infection.

It was important for our study to repopulate a decellularized xenograft scaffold with host cells in an *in vivo* environment. The analysis of long-term recellularization via an *in vivo* approach has been minimally studied [38]. It is often difficult to quantify the hemodynamic stresses that the aortic valve undergoes during the cardiac cycle in relation to recellularization [39]. Most studies have employed an *in vitro* approach where biological heart valve scaffolds are recolonized by autologous cells prior to implantation [38]. However, despite the widespread use of *in vitro* seeding, groups have begun to recolonize TEHVs using an *in vivo* approach because cell types specific to the host are available in a native homeostatic environment [26]. *In vivo* recellularization serves a greater purpose because it allows a xenograft to have the correct cell phenotype desired for successful cell colonizing.

Immunohistochemical staining was used to determine cell infiltration in the valve cusps. The two cell types observed in our study were valvular interstitial cells (VICs) and valvular endothelial cells (VECs). The antibodies that were utilized in this experiment reflected the observation of these specific cell types. VICs play an important role in maintaining the function of the valvular cusp that include the formation of new collagen [40]. In our histological sections, it was observed that there was a significant amount of interstitial-like cell infiltration found within the aortic cusps. Normal cardiac valves contain myofibroblast-like cells that compose a majority of the VIC population; however, the development and adaptation of these cells has yet to be entirely understood [40]. In our study, there was an apparent amount of myofibroblast-like VICs as indicated by positive α-SMA and vimentin expression. Cells with this phenotype are known to remodel the connective tissue found within the valve cusps. The natural hemodynamic forces activated VICs to create new collagen fibrils, which then impacted our explanted mechanical properties. This process of collagen formation due to physiological forces is similarly found during human fetal development [40]. Upon explant, mechanical testing results showed an increase in tissue stiffness of the cusps that was likely attributed to infiltration of myofibroblast-like cells.

Our recellularized valves showed cells originating from the ventricular side of our valve cusps. Recellularization occurred predominately near the cusp edge and infiltrated toward the outer portion of the cusps (ventricular side to the pulmonic side) indicating that the blood flow had a large role in the recellularization process. Previous reports have shown different recellularization locations that stem from the aortic side of the cusps [28]. However, the mechanism of recellularization in our study indicates that factors such as ECM porosity due to the decellularization process, hemodynamic pressures, and directional flow of blood may have influenced the location of *in vivo* regeneration. In addition, our group has concluded that the cells that are responsible for the recellularization of cusps actually come from the circulating blood. Studies done by Takagi *et al*. have hypothesized that progenitor cells found in the systemic circulation are responsible for the repopulation of VICs and the transdifferentiation from VECs into smooth muscle cells [26].

## Limitations

In order to progress in our studies, we have considered several variables that need further exploration. While this study observed implantation into the RVOT of sheep, future studies examining the feasibility of implantation into the aortic position will be carried out. It may be speculated that the aortic environment may be able to stimulate more VICs due to the high pressure found in the aorta. Although there has been extensive literature on VIC and VEC phenotypes regarding recellularization, we plan to observe the role of different macrophage phenotypes and their role in regeneration [24]. To minimize research on animals, *in vitro* accelerated wear testing and/or bioreactors systems may provide approximate valve durability. However, the *in vivo* regeneration response will still need to be evaluated with chronic animal studies. It will be necessary to establish an optimal decellularization process that minimizes weight to volume ratios, detergent concentrations, and processing time. This process optimization will ensure antigen removal while still preserving the ECM found in our scaffolds. Due to the availability of our samples, only the circumferential direction was considered during mechanical testing. However, for future experiments, biaxial testing will be considered to evaluate the anisotropy of the valve cusps.

## Conclusion

In summary, the current study demonstrated the feasibility of transpecies implantation of an off-the-shelf decellularized porcine aortic valve into the RVOT of sheep. The implantation resulted in recellularization of the valve with sufficient hemodynamic function for the 5-month study. Thus, the study supports a potential role for the use of a TEHV for the treatment of valvular heart disease in humans.

## Supporting information

S1 Checklist ARRIVE checklist. The ARRIVE Guidelines Checklist Animal Research: Reporting In Vivo Experiments.
(PDF)
